# The shell morphology of the latest Cretaceous (Maastrichtian) trionychid turtle *Helopanoplia distincta*

**DOI:** 10.7717/peerj.4169

**Published:** 2017-12-14

**Authors:** Walter G. Joyce, Tyler R. Lyson

**Affiliations:** 1 Departement für Geowissenschaften, Universität Freiburg, Freiburg, Switzerland; 2 Department of Earth Sciences, Denver Museum of Nature and Science, Denver, CO, United States of America

**Keywords:** Maastrichtian, Testudines, Trionychidae, Cryptodira, *Helopanoplia distincta*, Hell Creek Formation, *Plastomenidae*, Late Cretaceous, Lance Formation

## Abstract

**Background:**

*Helopanoplia distincta* is an extinct soft-shelled turtle (Pan-Trionychidae) for which the type specimen is a fragmentary costal and the inguinal notch portion of the left hypoplastron from the Late Cretaceous (Maastrichtian) Lance Formation of Wyoming, USA that bear a distinct surface sculpture pattern consisting of raised tubercles. Over the course of the past few decades, a number of additional, fragmentary specimens from the Late Cretaceous (Maastrichtian) Hell Creek Formation of Montana and North Dakota have been referred to this taxon based on the presence of these tubercles, but a more complete understanding of the anatomy and phylogenetic relationships of this distinctive soft-shelled turtle is still outstanding.

**Methods:**

We here figure and describe shell remains of eight fossils referable to *Helopanoplia distincta* from the Hell Creek Formation of Montana and North Dakota that, in combination, document nearly all aspects of the shell morphology of this taxon. We furthermore explore the relationships of this fossil turtle by inserting it into a modified phylogenetic analysis of pan-trionychid relationships.

**Results:**

The new fossil material thoroughly supports the validity of *Helopanoplia distincta*. In addition to its unique surface sculpture pattern, this turtle can be diagnosed relative to all other named pan-trionychids by the presence of a distinct corner along the margin of costals II, the complete covering of costal ribs I–VI by metaplastic bone, midline contact of the main plastral elements, hyoplastral shoulder, presence of a lateral, upturned margin on the hyo/hypoplastron that is covered dorsally and laterally by sculptured metaplastic bone, a single, lateral hyoplastral process, and the apomorphic presence of fine scallops along the margin of costals VIII, formation of a laterally embraced, rounded nuchal, anteriorly rounded costals I, distally expanded costals II, and narrow costals VII. A phylogenetic analysis places *Helopanoplia distincta* as sister to the clade formed by *Plastomenus thomasii* and *Hutchemys* spp., thereby confirming its identity as a plastomenid. The vast majority of *Helopanoplia distincta* material has been recovered from fine-grained overbank deposits, thereby supporting the hypothesis that this turtle favored ponded waters.

## Introduction

Pan-Trionychidae, soft-shelled turtles, is a diverse clade of turtles with an extensive fossil record that extends from the Early Cretaceous (Aptian) to the Recent. Although trionychids are currently restricted to Asia, North America, and Africa, fossils document their former presence in Australia, Europe, and South America as well (Vitek & Joyce, 2015; Georgalis & Joyce, 2017; Brinkman, Rabi & Zhao, 2017). Extant representatives of the clade are easily recognized externally by their flat shell covered by a leathery skin, well-developed, soft paddles, and an elongate proboscis (Ernst & Barbour, 1989). In addition, the skeletal morphology of the group is unique among turtles in having a reduced carapace that lacks peripherals and pygals and a restructured plastron consisting of strap-like epiplastra that lack a midline contact and a boomerang-shaped entoplastron that posteriorly embraces the hyoplastra (Meylan, 1987). The endoskeletal portions of the shell are covered by distinctly ornamented dermal bone with a unique microtexture consisting of layers of interwoven collagen fibers (Scheyer et al., 2007). Thus, even fragmentary fossil shell remains can easily be identified as pertaining to the group (e.g., Hutchison & Archibald, 1986).

The contemporaneous latest Cretaceous (Maastrichtian) Lance Formation and Hell Creek Formation of North Dakota, South Dakota, Montana, and Wyoming, USA, have yielded an exceptional turtle fauna that include numerous pan-trionychids (Holroyd, Wilson & Hutchison, 2014; Vitek & Joyce, 2015). Although many other turtle groups are known from these formations by complete shells or skulls that allow assessing their anatomy and phylogenetic relationships with more confidence (e.g., Riggs, 1906; Hay, 1908; Case, 1939; Whetstone, 1978; Meylan & Gaffney, 1989; Gaffney, 1972; Lyson & Joyce, 2009a; Lyson & Joyce, 2009b; Knauss et al., 2011), trionychids are still mostly known from fragments and their diversity can only be assessed by reference to shell fragments. A recent review of pan-trionychid material from the Lance and Hell Creek Formations concluded that at least eight lineages are present in these formations (Holroyd, Wilson & Hutchison, 2014) of which only three, “*Aspideretes*” *beecheri* Hay, 1904, *Axestemys splendida* (Hay, 1908), and *Gilmoremys lancensis* (Gilmore, 1916), are known to the scientific community by detailed figures of actual shells or crania (Hay, 1904; Joyce & Lyson, 2011; Vitek, 2012; Joyce et al., 2016).

**Figure 1:**
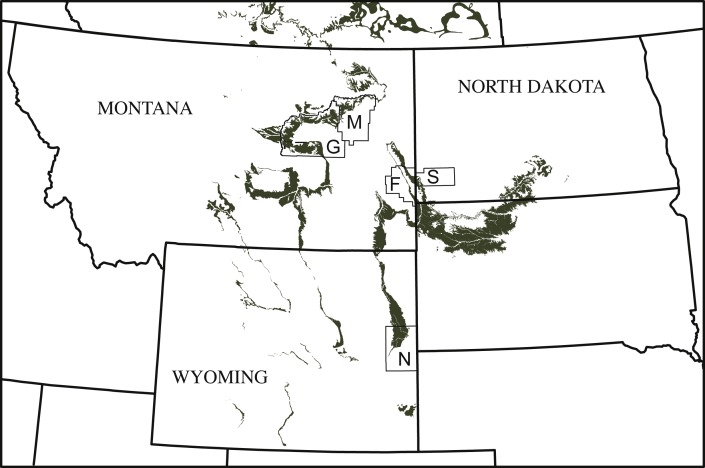
Simplified map of Montana, North Dakota, and Wyoming illustrating the known county distribution of *Helopanoplia distincta.* Outcrops of the Lance Formation and Hell Creek Formation are highlighted in grey. Abbreviations: *F*, Fallon County; *G*, Garfield County; *M*, McCone County; *N*, Niobrara County; *S*, Slope County.

One of the most enigmatic trionychids from the Lance and Hell Creek Formations is *Helopanoplia distincta* Hay, 1908. The type specimen of this species is two shell fragments, the distal portion of a costal and the inguinal notch portion of the left hypoplastron, which are externally decorated with distinct tubercles. Although the vast majority of fossil pan-trionychids based on equally scant type material are considered nomina dubia (Vitek & Joyce, 2015; Georgalis & Joyce, 2017), there is universal agreement that the surface sculpture of *Helopanoplia distincta* is indeed distinct, hence the species name, and diagnoses a valid taxon (Hutchison & Archibald, 1986; Holroyd & Hutchison, 2002; Holroyd, Wilson & Hutchison, 2014; Vitek & Joyce, 2015). Numerous fragments referred to this species from the Hell Creek Formation of Montana and North Dakota (Fig. 1) allow the shell of this species to be partially reconstructed (Holroyd & Hutchison, 2002, Fig. 2J), but many aspects of its anatomy remain unknown. The primary purpose of this contribution is to improve the understanding of *Helopanoplia distincta* by figuring and describing a series of previously published and unpublished fossils from the Hell Creek Formation that collectively provide a near complete understanding of its shell morphology. In addition, we assess the paleoecology of this species and explore its phylogenetic position through cladistic analysis.

## Systematic Paleontology

**Table:** 

TESTUDINES Batsch, 1788
PAN-TRIONYCHIDAE Joyce, Parham & Gauthier, 2004
PLASTOMENIDAE Hay, 1908

***Helopanoplia distincta*** Hay, 1908

Holotype—USNM 5732, a distal fragment of a costal and inguinal notch portion of the left hypoplastron (Hay, 1908, pl. 88, fig. 4, 5).

Type locality and horizon—Lance Creek, Niobrara County, Wyoming, USA; Lance Formation, Maastrichtian, Late Cretaceous (Hay, 1908). The type locality was originally reported by Hay (1908) to be located in Converse County, but this county was split in 1911 into a western (Converse) and an eastern (Niobrara) county. We infer that the type locality is located in Niobrara County, as the town of Lance Creek and the Lance Creek drainage are both located in this county.

**Figure 2:**
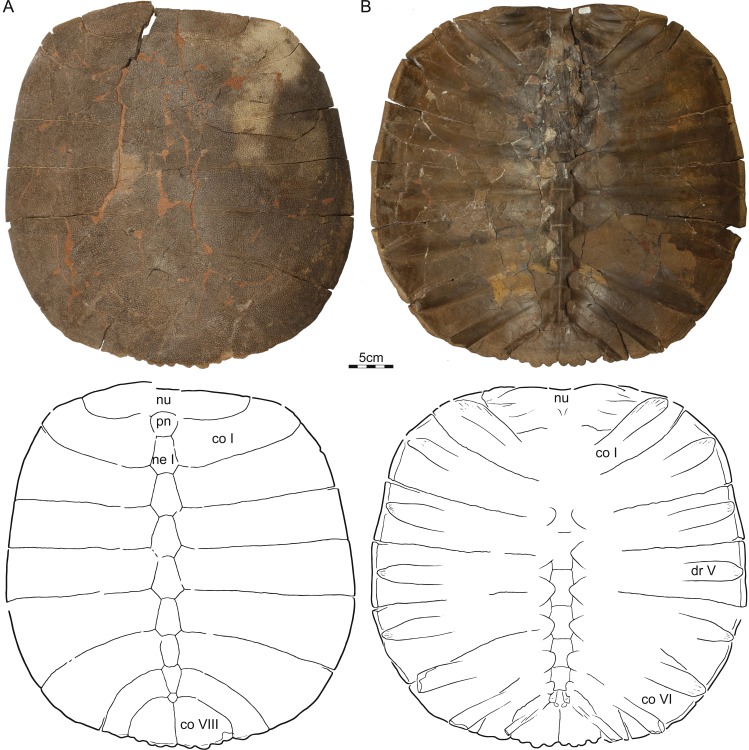
NDGS 353, *Helopanoplia distincta*, NDGS loc. 64, Slope County, North Dakota, USA, Hell Creek Formation, Late Cretaceous, Maastrichtian. Photographs and illustrations of carapace in (A) dorsal and (B) ventral view. Abbreviations: *co*, costal; *dr*, dorsal rib; *ne*, neural; *nu*, nuchal; *pn*, preneural.

Referred material—NDGS 353, NDGS loc. 64, a nearly complete carapace (Fig. 2), Hell Creek Formation, found in a soft, tan, finely bedded sandstone, collected within the upper 25 m of the Hell Creek Formation, Slope County, North Dakota, USA.

**Figure 3:**
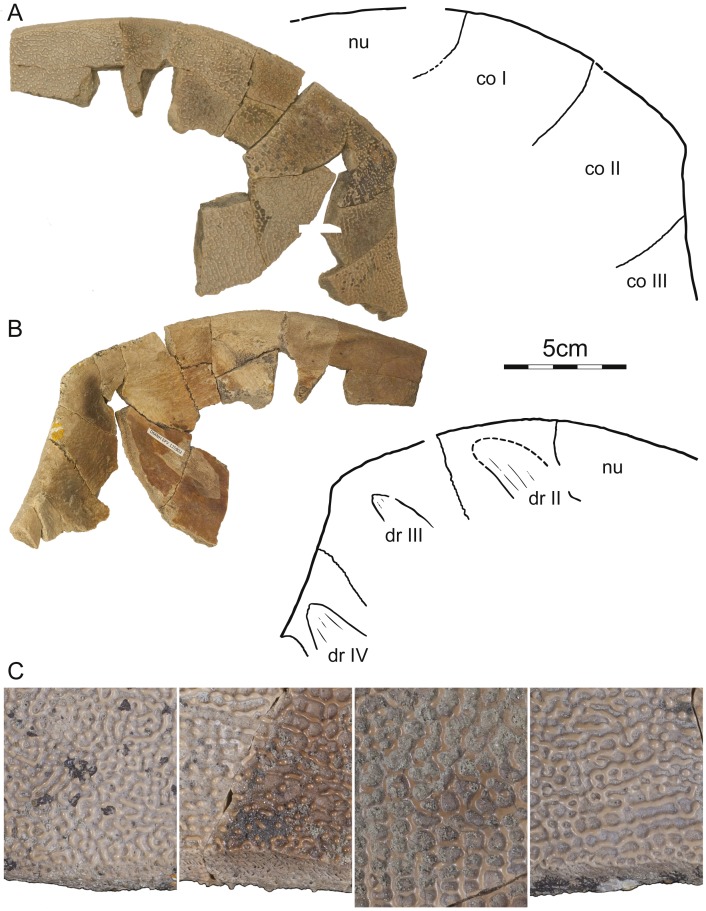
DMNH 125902, *Helopanoplia distincta*, DMNH loc. 7179, Fallon County, Montana, USA, Hell Creek Formation, Late Cretaceous, Maastrichtian. Photographs and illustrations of anterior right portions of the carapace in (A) dorsal and (B) ventral view. (C) Detailed photographs of shell fragments with uncertain identity highlighting variation in surface sculpture. Abbreviations: *co*, costal; *dr*, dorsal rib; *nu*, nuchal.

DMNH EPV.125902, DMNH loc. 7179, a fragmentary, disarticulated shell, including, among others, a partial right nuchal and the distal portions of right costals I–III (Fig. 3), Hell Creek Formation, found at the base of an approximately 2 m thick channel sandstone 7.5 m below the contact with the overlying Fort Union Formation, Fallon County, Montana, USA.

**Figure 4:**
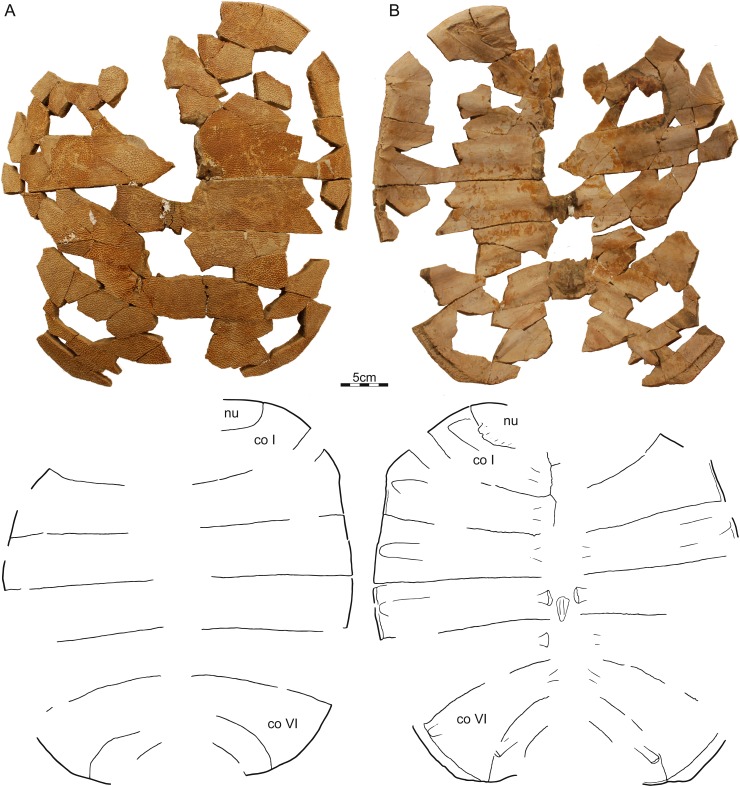
PTRM 2767, *Helopanoplia distincta*, PTRM loc. V86006, Slope County, North Dakota, USA, Hell Creek Formation, Late Cretaceous, Maastrichtian. Photographs and illustrations of carapace in (A) dorsal and (B) ventral view. Abbreviations: *co*, costal; *ne*, neural; *nu*, nuchal; *pn*, preneural.

PTRM 2767, PTRM loc. V86006, a fragmented, partial carapace (Fig. 4), Hell Creek Formation, found in a soft sandstone channel approximately 33.65 m below the contact with the overlying Fort Union Formation, Slope County, North Dakota, USA.

**Figure 5:**
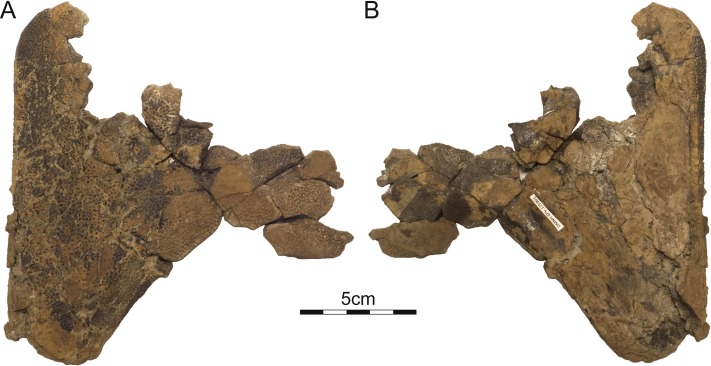
DMNH 125901, *Helopanoplia distincta*, DMNH loc. 5873, Slope County, North Dakota, USA, Hell Creek Formation, Late Cretaceous, Maastrichtian. Photographs of partial right hyo/hypoplastron in (A) ventral and (B) dorsal view.

DMNH EPV.125901, DMNH loc. 5873, a partial right hyo/hypoplastron (Fig. 5), Hell Creek Formation, collected from an iron rich siltstone 13 m below the contact with the overlying Fort Union Formation, Slope County, North Dakota, USA.

**Figure 6:**
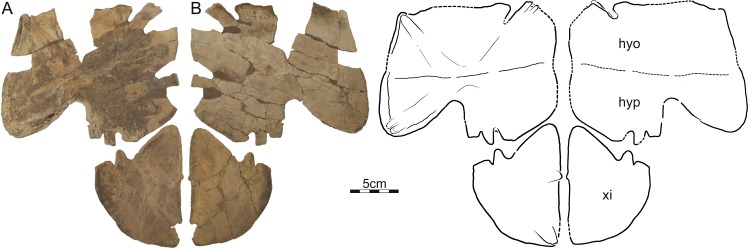
DMNH 125900, *Helopanoplia distincta*, DMNH loc. 5853, Fallon County, Montana, USA, Hell Creek Formation, Late Cretaceous, Maastrichtian. Photographs and illustrations of partial plastron in (A) dorsal and (B) ventral view. Abbreviations: *hyo*, hyoplastron; *hyp*, hypoplastron; *xi*, xiphiplastron.

DMNH EPV.125900, DMNH loc. 5853, a nearly complete left hyo/hypoplastron and left xiphiplastron (Fig. 6), Hell Creek Formation, recovered from a hard siltstone 17 m below the overlying contact with the Fort Union Formation, Fallon County, Montana, USA.

**Figure 7:**
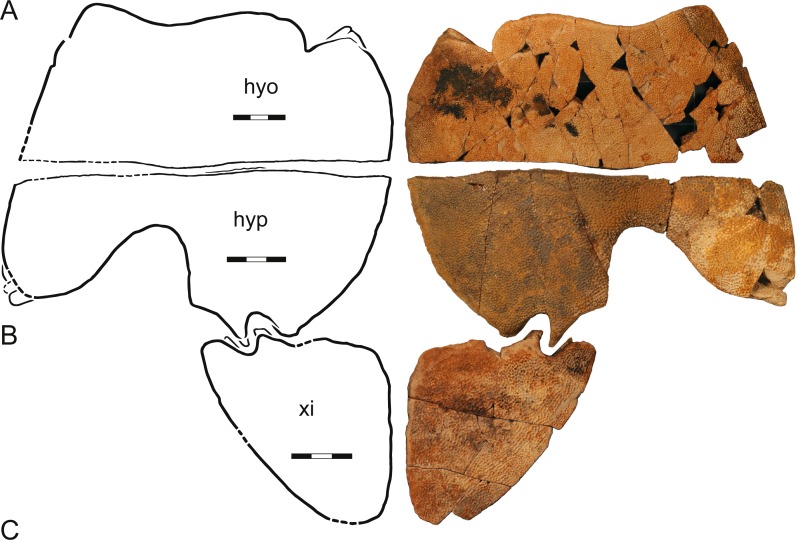
A composite plastron, *Helopanoplia distincta*, Garfield and McCone Counties, Montana, USA, Hell Creek Formation, Late Cretaceous, Maastrichtian. Photographs and illustrations of (A) a left hyoplastron (UCMP 131614, UCMP loc. V84187), (B) a left hypoplastron (UCMP 130112, UCMP loc. V79116), and (C) a left xiphiplastron (UCMP 130113, UCMP loc. V79116). The illustrations are mirrored to highlight outline of plastron. Note that each element has a different scale bar. Abbreviations: *hyo*, hyoplastron; *hyp*, hypoplastron; *xi*, xiphiplastron.

Of the long list of fragments previously referred to *Helopanoplia distincta* based on their unique sculpturing (e.g., Hutchison & Archibald, 1986; Holroyd & Hutchison, 2002; Holroyd, Wilson & Hutchison, 2014), we here (Fig. 7) four particularly informative specimens, which also served as the basis for the reconstruction of *Helopanoplia distincta* provided by Holroyd & Hutchison (2002):

UCMP 130112 and 130113, UCMP loc. V79116, a left hypoplastron and left and right xiphiplastra, respectively, Hell Creek Formation, Garfield County, Montana, USA.

UCMP 131614, UCMP loc. V84187, a left hyoplastron, Hell Creek Formation, McCone County, Montana, USA.

UCMP 134502, UCMP loc. V87080, an assortment of shell fragments including left costals VI and VII, Hell Creek Formation, McCone County, Montana, USA.

Distribution—Late Cretaceous (Maastrichtian), Lance Formation of Niobrara County, Wyoming (Hay, 1908) and Hell Creek Formation of Fallon, Garfield, and McCone counties, Montana, and Slope county, North Dakota, USA (Hutchison & Archibald, 1986; Holroyd & Hutchison, 2002; Holroyd, Wilson & Hutchison, 2014; and new material described herein; Fig. 1).

Diagnosis—*Helopanoplia distincta* can be diagnosed as a pan-trionychid, among others, by the absence of peripherals, pygals, and scutes. *Helopanoplia distincta* can be distinguished from all other known pan-trionychids by the development of distinct tubercles along the margins of the carapace and much of the plastron. *Helopanoplia distincta* can furthermore be distinguished from all other currently recognized Late Cretaceous to Paleocene pan-trionychids from North America by the following combination of characters: formation of a distinct corner along the margin of costals II (also seen in some individuals of *Hutchemys sterea*), the apomorphic formation of fine scallops along the margin of costals VIII, the complete covering of costal ribs I–VI (i.e., dorsal ribs II–VII) by metaplastic bone (also seen in *Atoposemys superstes*), the apomorphic formation of a laterally embraced, rounded nuchal, anteriorly rounded costals I, distally expanded costals II, narrow costals VII, a blunt midline contact of the main plastral elements (also present in *Hutchemys* spp.), hyoplastral shoulders (also present in most other plastomenids), presence of an upturned margin formed by the hyo/hypoplastron that is covered dorsally by sculptured metaplastic bone (also present in *Hutchemys* spp.), and a single, anterolateral hyoplastral process (also present in *Axestemys* spp. and *Hutchemys* spp.).

## Description

Carapace—The carapace of *Helopanoplia distincta* is rounded in outline, with exception of a blunt (Fig. 2) to distinct (Figs. 3 and 4) shoulder formed by costals II and scalloping along the posterior margin of costals VIII (Fig. 2). NDGS 353, the only articulated specimen, is approximately 39 cm long along the midline and approximately 38 cm wide at the suture of costals IV and V (Fig. 2). The carapace of DMNH EPV.125902, a disarticulated specimen of similar size, varies in thickness from 6 mm near the midline to 11 mm towards the limits of costals II–VI, not including the ribs. The margins of the carapace are generally rounded and only show minor evidence of “splitting” along the distal edge (i.e., a trough along the margin that separates the most surficial layers from the deepest layers of the costal, see Joyce et al., 2009 for figures) along the thickened margins of costals II–VI. Metaplastic ossification fully covers costal ribs I–VI (= dorsal ribs II–VII). The majority of the carapace is covered with an irregularly netted sculpturing pattern with thickened tubercles that decorate the intersections of some ridges (Fig. 2). A sculpturing pattern consisting of isolated tubercles is only weakly apparent in the nuchal region of NDGS 353 and PTRM 2767 (Figs. 2 and 3), but succinctly developed along the margins of the nuchal and costal I–III of DMNH EPV.125902 (Fig. 4).

In dorsal view, the nuchal is a broad, rounded element that is about six times broader than long and is laterally braced by the costals. Metaplastic ossification fully covers the cleithral portions (sensu Lyson et al., 2013) of the bone. A small, posterior embayment of the nuchal receives the preneural. The full neural column is only preserved in NDGS 353. In this specimen, the column consists of a preneural and eight neurals, which prevent a midline contact of all costals except costals VIII. The preneural is a rounded element that is about as wide as long, approximately the length of neural VII, and forms an anteriorly convex contact with the nuchal. While neurals I–V are hexagonal and have short posterior sides, neural VI has the shape of a rounded rectangle, neural VII is hexagonal with short anterior sides, and neural VIII is notably small and pentagonal. The arrangement of the posterior neurals is slightly different from that reported by Holroyd & Hutchison (2002), but this is attributable to intraspecific variation (see Discussion below). Eight pairs of costals are present. Costals I are strongly bowed anteriorly and thereby surround the nuchal anteromedially. Costals II mediate between the anteriorly bowed costals I and the straight costals III and therefore strongly expand distally. At mid-length, the margin of costals II form a shoulder that ranges from blunt in NDGS 353 (Fig. 2) to distinct in DMNH EPV.125902 (Fig. 3) and PTRM 2767 (Fig. 4). Costals III and IV are the proximodistally longest costals and have parallel margins. Costals V and VI are slightly bowed towards the posterior and slightly expand distally. While costals I–VI have similar anteroposterior dimensions near their contacts with the neurals, costal VII is significantly shorter. Costal VIII is triangular and has anteroposterior proportions more reminiscent of costals I–VI. NDGS 353 only preserves a midline contact for costals VIII (Fig. 2), which stands in contrast to the midline contact of costals VII and VIII reported by Holroyd & Hutchison (2002).

The deep, cleithral portions of the nuchal form a coarse comb of lateral processes in ventral view that is fully covered by metaplastic bone dorsally and partially covered by the expanded dorsal rib II in ventral view. Dorsal vertebrae III–IX are preserved in varying quality in NDGS 353. The available centra are flattened, have broad contacts with one another and have interdigitated contacts with the adjacent ribs. Dorsal rib I is not preserved in any specimen. Dorsal rib II (= costal rib I), best preserved in NDGS 353, is only modestly expanded distally, ends bluntly, partially covers the nuchal comb ventrally, and is fully covered by metaplastic bone dorsally. Dorsal ribs III–VI (= costals ribs II–V) also expand only lightly distally, have pointed tips, and are fully covered dorsally by metaplastic bone. Dorsal rib VII (= costal rib VI) is broad in NDGS 353 and slightly underlaps costals V, but those of PTRM 2767 are narrower and do not underlap costals V. This rib, however, is fully covered by metaplastic bone in both specimens. Dorsal ribs VIII and IX (= costal ribs VII and VIII) are not fully preserved in any specimen. However, the clear breaks apparent at the posterior margins of NDGS 353 and PTRM 2767 indicate that dorsal ribs VIII are significantly narrower that the more anterior ones, clearly underlap costals VI, and protruded beyond the margin of the carapace. Dorsal ribs IX are even narrower, clearly underlap costal VII, and likely protruded significantly beyond the posterior margin of the carapacial disk.

Plastron—Only the hyoplastron, hypoplastron, and xiphiplastron are known for *Helopanoplia distincta* (Figs. 5–7). Whereas much of the hyo- and hypoplastron are decorated with the distinct tubercles that diagnose *H. distincta*, the xiphiplastron is covered by a broad, netted pattern more reminiscent of the carapace (Figs. 5–7). Ornamented, metaplastic bone is furthermore apparent on the dorsolateral side of the hyo- and hypoplastron in larger specimens (Figs. 5–7). The hyo- and hypoplastron are sutured to one another, but apparently only resisted disarticulation after death in the largest individuals (Figs. 5 and 6). The fusion of the hyo/hypoplastron during ontogeny is a common feature among extant trionychids (Meylan, 1987).

The strap-like processes that form the deep tissue component of the available plastral material is nearly fully hidden in ventral view by metaplastic bone. The hyoplastron has a single lateral process and two medial processes only. The hypoplastron has two lateral processes, a single, medial process, and two posterior processes. The xiphiplastron has two clear anterior processes and two medial processes.

The metaplastic portions of the hyoplastron have a rounded to flattened medial margin, but while it is clear that a midline contact was absent in smaller specimens (Fig. 7), it is unclear if such a contact was present in large specimens (Fig. 6). Although the entoplastron is unknown, the hyoplastron forms a well-developed anterior lappet that appears to have partially (Fig. 7) or completely (Fig. 6) restricted mobility of the entoplastron. The medial margin of the hypoplastron is rounded to flattened (Figs. 5 and 6), but it is unclear once again if a midline contact was present in larger specimens. The posterior aspects of the hypoplastron are deeply incised for contact with the xiphiplastron. This contact was strengthened by a bony flap formed lateral to the posterior plastral processes. Metaplastic bone not only covers the ventral aspects of the posterior plastral lobe, but also wraps onto its posterior aspects. The hyo- and hypoplastra combined form an upturned lip at their lateral margins that rises towards the midline at an angle of 70 degrees and that is decorated on its dorsal side with sculptured metaplastic bone (Figs. 6 and 7). In life, this would have represented the lateral margin of the animal. The xiphiplastra form expanded metaplastic callosities that likely contacted one another along the midline in life, but show now evidence of midline suturing. The lateral margin is broadly rounded, protruding far beyond the margin of the deep tissue processes. Whereas a large fontanelle appears to have been present between the hypo- and xiphiplastra in medium sized individuals (Fig. 7), this space is filled by an expanded anterior process of the xiphiplastra in the largest available specimen (DMNH 125900; Fig. 6).

## Phylogenetic Analysis

### Matrix

We explore the phylogenetic relationships of *Helopanoplia distincta* by inserting it into the character taxon matrix of Brinkman, Rabi & Zhao (2017), which in turn is based on the matrix of Meylan (1987) with modifications from Joyce et al. (2009), Joyce et al. (2016), Joyce & Lyson (2011) and Li, Joyce & Liu (2015). We generally follow the modifications of Brinkman, Rabi & Zhao (2017) to the matrix of Joyce et al. (2016), with the following exceptions:

1. We reinsert *Atoposemys superstes* following Joyce et al. (2016).
2. Character 4 (presence of preneural): We score *Kuhnemys maortuensis* “2” (preneural absent) not “?” based on photographs of the holotype (IVPP 2864).
3. Character 7 (size of costal VIII): We score *Petrochelys kyrgyzensis* “?” not “1”, as the relevant part of the shell is not preserved (Nessov, 1995). We furthermore score *Kuhnemys maortuensis* “2” (costals VIII small) not “1” based on photographs of the holotype (IVPP 2864).
4. Character 14 (extended midline contact of xiphiplastral callosities): Meylan (1987) developed a character that pertains to the fusion of the xiphiplastra along the midline, but the wording is imprecise, as these bones are not actually fused in any specimens available to us, but rather form a blunt, at best sutural contact along the midline. We therefore rephrase this character for greater clarity.
5. Characters 8–12 (presence of plastral callosities): Meylan (1987) developed a character that counts the number of plastral callosities in the plastron of trionychids. Although this character can easily be scored for extant trionychids for which the entire skeleton is known, its application is more complicated among fossils, as complete plastra are rarely preserved. We therefore here translate Meylan’s (1987) original character into five separate characters that capture the absent (0) or presence (1) of epiplastral (character 8), entoplastral (character 9), hyo/hypoplastra (character 10), xiphiplastral (character 11), and supernumerary (character 12) callosities. As the vast majority of trionychids have at least some bony ossification developed along the hyo-, hypo-, and xiphiplastra, we here only score callosities as present for these bones if they exhibit well-developed surface sculpturing. The majority of Mesozoic forms included herein are therefore scored as lacking callosities for most bones. *Gilmoremys lancensis* is scored as possessing an entoplastral callosity based on UCMP 129185. All outgroups were scored inapplicable for these characters.
6. Character 16 (neural number): We favor not including the preneural in the neural count and in using polymorphic characters in contrast to polymorphic characters states. We therefore utilize the character definitions and scorings of Joyce et al. (2016) for this character.
7. Character 17 (midline contact of costals): We score *Petrochelys kyrgyzensis* “?” not “1”, as the relevant portion of the shell is not preserved (Nessov, 1995). We furthermore score *Kuhnemys maortuensis* “0” (midline contact absent) not “1”, based on photographs of the holotype (IVPP 2864).
8. Character 19 (placement of neural reversal): As with character 16, we prefer using polymorphic character states and we therefore reverted this character to the definitions and scoring of Joyce et al. (2016). We furthermore score *Kuhnemys maortuensis* 2 not 3 (i.e., reversal at neural VI) by reference to photographs of the holotype (IVPP 2864).
9. Character 20 (suprascapular fontanelles): We note that Brinkman, Rabi & Zhao (2017) change the scoring of *Perochelys lamadongensis* from “1” to “2/3” (e.g., 2 or 3), not “3” as stated in the text, and we therefore amend this error. We similarly change the scoring of *Kuhnemys maortuensis* from “?” to “3” (suprascapular fontanelles open throughout life) based on photographs of the holotype (IVPP 2864) and that of *Petrochelys kyrgyzensis* from “1” to “3” based on Nessov (2005, fig. 3l).
10. Character 21 (epiplastral shape): We change the scoring of *Hutchemys rememdium* from “?” to “1” by reference to Hutchison (2009), as *Plastomenoides lamberti* is the objective junior synonym of *Hutchemys rememdium* (Vitek & Joyce, 2015).
11. Character 22 (epiplastral length): We change the scoring of *Plastomenus thomasii*, *Hutchemys rememdium*, and *Hutchemys sterea* from “?” to “1” (epiplastra short) by reference to Hutchison (2009). We furthermore change the scoring of *Gobiapalone orlovi*, *Perochelys lamadongensis*, and *Petrochelys kyrgyzensis* from “2” to “1”, as all possess epiplastra that Meylan (1987) would have considered to be short, not intermediate.
12. Character 24 (bridge length): We score *Kuhnemys maortuensis* as “2” (bridge short) not “?” by reference to pictures of the holotype (IVPP 2864).
13. Character 28 (jugal squamosal contact): We return the scoring of *Perochelys lamadongensis* from “2” to “?” as the relevant region is not preserved in the only known specimen (Li, Joyce & Liu, 2015).
14. Character 29 (jugal parietal contact): As for character 16, we return the scoring to binary scoring, not polymorphic.
15. Character 39 (closing of foramen jugulare posterius): Brinkman, Rabi & Zhao (2017) accidentally reverted the scoring of this character back to that of one of its sub-characters as originally scored by Meylan (1987). As a result, *Dogania subplana*, *Palea steindachneri*, *Pelodiscus sinensis* were score as lacking an enclosed posterior jugular foramen. We amend this mistake.
16. Character 40 (bones that contribute to the closing of foramen jugulare posterius): This character was removed from the matrix by Brinkman, Rabi & Zhao (2017) without justification. We include it again.
17. Characters 44, 48, 51: As with character 16, we return the scoring of these characters to exclude polymorphic character states.
18. Character 72 (nuchal notch): We rescore *Hutchemys arctochelys* “1” (nuchal notch present), not “2”, by reference to Joyce et al. (2009). We furthermore score *Atoposemys superstes* as “1”, not “?” by reference to Hutchison (2013).
19. Character 74 (split costals): We rescore *Plastomenus thomasii* as “1” (split costals present), not “0” by reference to AMNH 6018.
20. Character 79 (mobility of entoplastron): We rescore *Aspideretoides foveatus* and *Atoposemys superstes* polymorphic “0&1”, not “1”, to highlight that this character changes in ontogeny, instead of just “1” by reference to TMP 98.12.24 for *Aspideretoides foveatus* and DMNH EPV.125907 for *Atoposemys superstes*.
21. Character 80 (peripheral ossification): We rescore *Hutchemys sterea* “1” (peripheral ossification present) not “0” by reference to Hutchison (2009) and DMNH EPV.125906.
22. Character 81 (number of hyoplastral processes): We rescore *Hutchemys sterea* “1” (one process present) not “?” by reference to DMNH EPV.125906.
23. Character 87 (proportions of costals VIII): We rescore *Hutchemys tetanetron* “0” (broader than long) not “?” by reference to Hutchison (2009).
24. Character 89 (medial hyoplastral processes): We rescore *Hutchemys sterea* and *Atoposemys superstes* “1” (strongly serrated medial processes absent) not “?” by reference to DMNH EPV.125906 and DMNH EPV.125907, respectively.

We finally add two new characters to the matrix:

25. Character 93: Metaplastic ossification covering the posterior margins of the lateral processes of the hypoplastron: 0 = absent; 1 = present, as in *Plastomenus thomasii*.
26. Character 94: Metaplastic ossification covering the tips of the ribs: 0 = all ribs have free ends that protrude beyond the margin of the carapacial disk; 1 = costal ribs I–VI completely covered by metaplastic bone, only costal ribs VII and VIII protrude beyond the margin of the carapace; 2 = all costal ribs completely covered by metaplastic bone.

The final character/taxon matrix consists of 94 characters for 36 taxa. The matrix, including all character state definitions, is provided in File S1.

### Analysis

We performed a parsimony analysis using the software TNT (Goloboff, Farris & Nixon, 2008). In our primary analysis, all characters were left at equal weight and characters 1, 3, 5, 14, 20, 22, 41, 54, 79, 81, and 94 were run ordered. The molecular topology of Le et al. (2014) was used to constrain the relationships of all extant trionychids, but fossil trionychids were allowed to float. The matrix was subjected to 1,000 replicates of random addition sequences followed by a second round of tree bisection-reconnection. The recent study of Goloboff, Torres & Arias (2017) concluded that phylogenetic analyses using parsimony may retrieve better results using implied weighting. For exploratory purposes, we therefore ran the analysis with implied weights ranging from 1 to 12 at full integers.

## Results

**Figure 8:**
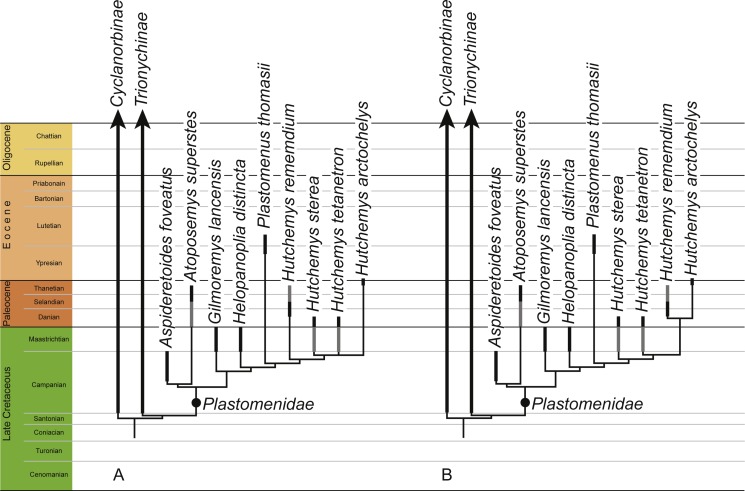
Phylogenetic hypothesis of plastomenid turtles. Time-calibrated strict consensus cladograms retrieved from the phylogenetic analysis using default weighting (A) and implied weighting with a *k* value of 11 (B). The full trees are provided in File S2.

Our primary phylogenetic analysis retrieved 56 equally parsimonious trees with 312 steps, a consistency index of 0.375 and a retention index of 0.608. The full strict consensus tree and a list of common synapomorphies are provided in File S2. An extraction of the tree relevant to this study is provided in Fig. 8A. Mild weighting with a *k* value of 11 results in 21 most parsimonious trees with a best score of 13.55014 and a novel arrangement within *Hutchemys*, which is more concordant with the stratigraphic record by implying fewer ghost lineages (Fig. 8B). The full tree is provided in File S2. In contrast to some previous analyses (e.g., Joyce et al., 2009; Joyce & Lyson, 2010; Joyce & Lyson, 2011; Li, Joyce & Liu, 2015), plastomenids are retrieved as stem-trionychines, regardless of the weighting applied, a conclusion also recently obtained by Brinkman, Rabi & Zhao (2017). The Early Cretaceous Asiatic pan-trionychids included in the matrix are retrieved as a clade in all analyses (File S2), but only extreme weighting with a *k* value of 1 retrieves this outside of crown Trionychidae. Incidentally, if the matrix is run without the molecular backbone constraint, these turtles are retrieved outside crown Trionychidae even without the application of weights.

## Discussion

### Alpha taxonomy

*Helopanoplia distincta* is based on just two shell fragments from the Late Cretaceous (Maastrichtian) Lance Formation of Wyoming (Hay, 1908), but its validity has never been challenged (e.g., Hutchison & Archibald, 1986; Holroyd & Hutchison, 2002; Holroyd, Wilson & Hutchison, 2014; Vitek & Joyce, 2015), because no other North American pan-trionychid turtle is known to possess a surface sculpturing consisting of distinct tubercles. Although numerous fragments had been referred to *Helopanoplia distincta* over the course of the last three decades (Hutchison & Archibald, 1986; Holroyd & Hutchison, 2002), these contributed little to a deeper understanding of the skeletal morphology of this species.

We here refer three partial to complete carapaces (Figs. 2–4) from the Hell Creek Formation of Montana and North Dakota to *Helopanoplia distincta* that document nearly all aspects of carapacial anatomy and help further support the validity of this species. Although all three specimens display the unique surface texture of *Helopanoplia distincta* along the margins of the nuchal and the anterior costals, the remaining parts of the shell are decorated with a more regular, netted pattern, that may be confused with that of *Axestemys splendida* (Vitek, 2012) or “*Aspideretes*” *beecheri* (Hay, 1908), two other pan-trionychids that also occur in the Hell Creek Formation. The distal ends of costal ribs I–VI (= dorsal ribs II–VII) are covered by metaplastic bone of the costals, but costal ribs VII and VIII (= dorsal ribs VIII and IX) protrude from the margin of the shell, a condition similar to that observed in *Atoposemys superstes* (= *Atoposemys entopteros* of Hutchison, 2013). As in the majority of other plastomenids, the margins of the costals are split, but the posterior margin of the shell is different by being convex and weakly scalloped. A distinct corner is developed along the margin of costal II, as in some individuals of *Hutchemys sterea* (DMNS EPV.125906). As in the majority of plastomenids, the nuchal is broad and the cleithral processes are fully covered by bone, but the nuchal is apomorphically surrounded by the anteriorly directly costals I in *Helopanoplia distincta*. Whereas costals II are greatly expanded distally, much as in *Gilmoremys lancensis* (Joyce & Lyson, 2011), costals VII are unique among plastomenids by being narrow. Of the three specimens available to us, only a single specimen (NDGS 353) documents the neural column as consisting of eight neurals (excluding the preneural) and as having a reversal at neural VI. This contrasts with UCMP 134502, a specimen referred to *Helopanoplia distincta* by Holroyd & Hutchison (2002), which only includes the left costals VI and VII, but implies a neural column with seven neurals and a reversal at neural VI, which is the more usual arrangement in plastomenids (e.g., Hutchison, 2009; Hutchison, 2013; Joyce et al., 2009). Additional specimens are needed to clarify to what degree *Helopanoplia distincta* is polymorphic in regards to its neural formula, but we speculate by reference to other plastomenids that the apomorphic arrangement documented by our material is the less common condition. Holroyd & Hutchison (2002) had previously noted in their composite illustration of the plastron of *Helopanoplia distincta* that the main plastral elements contact one another along the midline and that the hyoplastra formed broad shoulders (i.e., an anteriorly protruding lobe of metaplastic bone) that partially locked the entoplastron. We here note further that the hyoplastron only has a single medial process, much as representatives of *Hutchemys* spp., that the hyo/hypoplastron laterally develop metaplastic bone with surface sculpturing on their lateral and dorsal side, as in some *Hutchemys sterea* (Hutchison, 2009) and *Hutchemys arctochelys* (Joyce et al., 2009), and that the xiphiplastron fully fills the fontanelle between the hypoplastra. The epiplastra and entoplastron remain unknown. The long list of characters provided herein help to distinguish *Helopanoplia distincta* from all other known turtles from the Late Cretaceous to Paleogene of North America and thereby further supports the validity of this taxon.

### Phylogenetic relationships and paleoecology

Our analyses universally retrieve *Helopanoplia distincta* as sister to the clade formed by *Plastomenus thomasii* and *Hutchemys* spp within the clade Plastomenidae (Fig. 8). The initial assessment of Holroyd & Hutchison (2002) and Vitek & Joyce (2015) based on less complete material is therefore confirmed within a cladistic context. Characters that support this placement include the combined presence of a wide nuchal (character 1, also found in some trionychines), an extended midline contact of the xiphiplastra (character 14, also found in some cyclanorbines), the presence of seven neurals (character 15, also present in other trionychids), large adult size (character 24, also present in other trionychids), and ossification of the posterior margins of the lateral processes of the hypoplastron (character 93, also found in some cyclanorbines). Future finds will test whether this taxon possesses characters more unique to the group, such as the development of a rounded entoplastron (character 78) or various aspects pertaining to the development of broad triturating surfaces (characters 82–86). The conclusion that *Helopanoplia distincta* is a plastomenid further solidifies the notion the trionychid assemblage of Laramidia (i.e., the western portions of North America) was dominated by representatives of this clade during the Late Cretaceous (Vitek & Joyce, 2015).

Goloboff, Torres & Arias (2017) recently suggested that cladistic analyses using parsimony retrieve better results when mild implied weighting is implemented. Using implied weighting, Brinkman, Rabi & Zhao (2017) were recently able to retrieve Early Cretaceous pan-trionychids in a position outside of the crown group, but this result was only obtained when severe weighting with a *k* factor of 3 was applied, as originally suggested by Goloboff (1993). In our analysis, mild weighting with a *k* factor of 11 retrieves a topology that is slightly different from that obtained without weights, by suggesting that Paleocene representatives of *Hutchemys* are each other’s closest relatives (Fig. 8). As we only utilize mild weighting and as this result is more congruent with the fossil record by implying fewer ghost lineages, we find this result to be more plausible. Interestingly, even though we retrieve various Early Cretaceous pan-trionychids outside of crown Trionychidae when running the analyses within the molecular backbone constraint, only extremely heavy weighting using a *k* factor of 1 recovers this result (File S2). If all terminals in our analysis are assumed to be monophyletic, the phylogeny resulting from mild weighting implies that the latest Cretaceous (Maastrichtian) plastomenid fauna consisted of seven plastomenids, of which two are ghost lineages (i.e., the ancestral leading to *Atoposemys superstes* and *Plastomenus thomasii*). Of these seven lineages, two are currently thought to go extinct across the K/T boundary: *Gilmoremys lancensis* and *Helopanoplia distincta*. The realization that *Gilmoremys lancensis* is a valid taxon is still relatively new (Joyce & Lyson, 2011) and we therefore still think it to be plausible that a renewed study of early Paleocene material may reveal this taxon to be a survivor as well, as already noted by Holroyd, Wilson & Hutchison (2014). The conclusion that *Helopanoplia distinct* went extinct across the K/T boundary, on the other hand, is well supported by the distinct surface sculpture of this taxon in combination with extensive sampling across the boundary section (Hutchison & Archibald, 1986; Holroyd & Hutchison, 2002; Pearson et al., 2002; Holroyd, Wilson & Hutchison, 2014).

Holroyd & Hutchison (2002) noted that fragments of *Helopanoplia distincta* more commonly occur in mudstones/siltstones than in sandstones, which suggests that this turtle may have preferred ponded environments. We note that most of the material herein referred was collected from mudstones as well, therefore broadly confirming this conclusion.

##  Supplemental Information

10.7717/peerj.4169/supp-1 File S1 Character taxon matrix used in phylogenetic analysis, including full character list and character state definitions and backbone constraint tree

10.7717/peerj.4169/supp-2 File S2 Complete strict consensus and majority consensus topologies retrieved from the unweighted and weighted analysis and list of common synapomorphies from the unweighted analysis

## Institutional abbreviations

AMNH: American Museum of Natural History, New York, USA
DMNH: Denver Museum of Nature and Science, Denver, Colorado, USA
NDGS: North Dakota Geological Survey at the North Dakota Heritage Center and State Museum, Bismarck, North Dakota, USA
PTRM: Pioneer Trails Regional Museum, Bowman, North Dakota, USA
TMP: Tyrrell Museum of Paleontology, Drumheller, Alberta, Canada
UCMP: University of California Museum of Paleontology, Berkeley, California, USA
USNM: National Museum of Natural History, Smithsonian Institution, Washington, DC, USA

## Additional Information and Declarations

### Data Availability

The following information was supplied regarding data availability: The code is included in the Supplementary File.
