# Supplementary material for: The shell morphology of the latest Cretaceous (Maastrichtian) trionychid turtle Helopanoplia distincta
Source: PeerJ. 2017 Dec 14;5:e4169. doi: 10.7717/peerj.4169 (PMC5733369; doi:10.7717/peerj.4169)
Supplement: File S2 [file peerj-05-4169-s002.pdf]

Strict consensus topology obtained from unweighted analysis. Clade numbers are used below (see common synapomorphies).

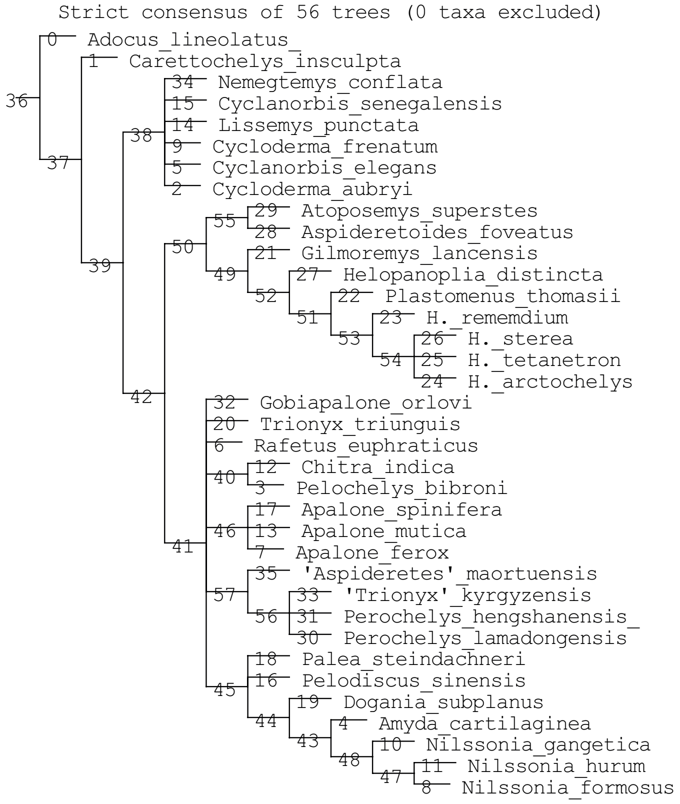

Majority rule consensus topology obtained from unweighted analysis. Numbers highlight the percentage of trees retrieving a particular clade above 50%.

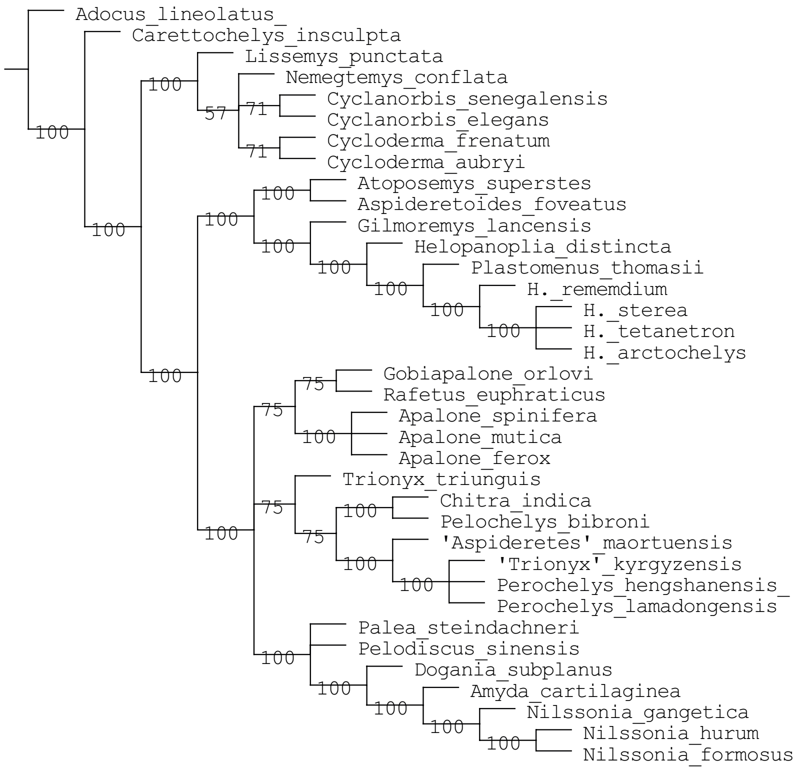

Strict consensus topology obtained from weighted analysis (k factor = 11).

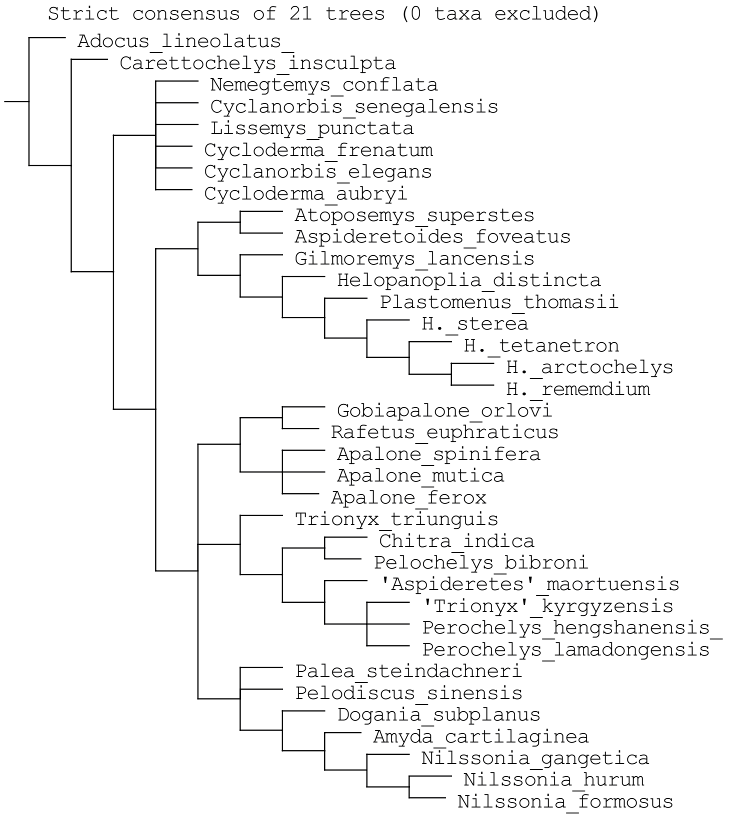

Majority rule consensus topology obtained from weighted analysis. Numbers highlight the percentage of trees retrieving a particular clade above 50%.

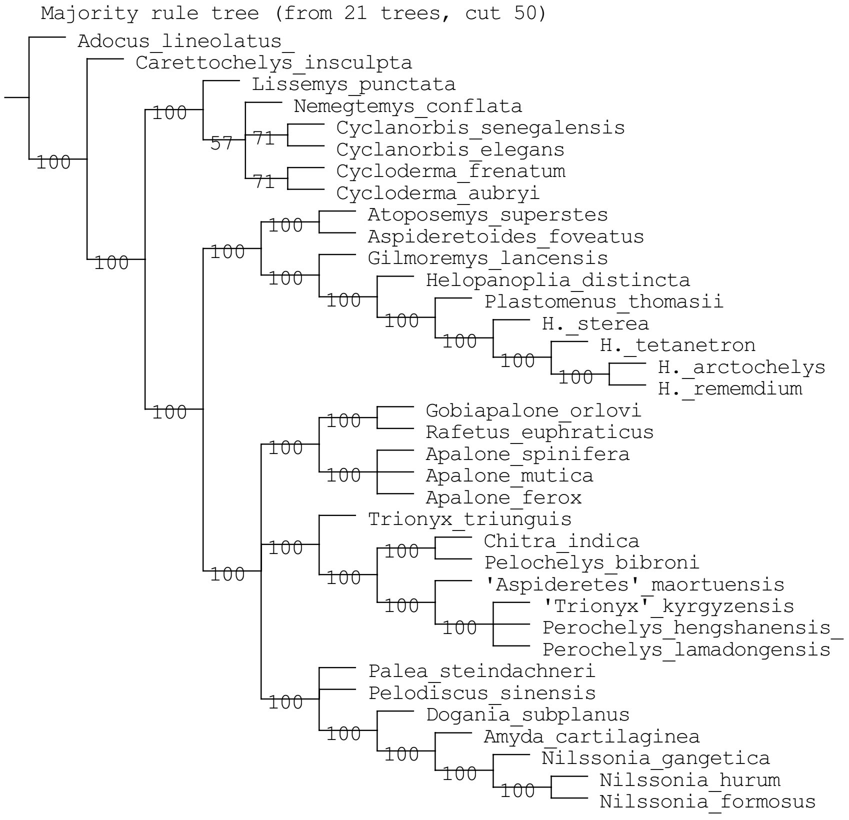

Synapomorphies common to 56 trees

Node numbers refer to nodes in consensus. Characters are numbers starting with 0, not 1.

Adocus_lineolatus_ :

All trees:

No autapomorphies:

Carettochelys_insculpta :

All trees:

Char. 17: 2 --> 3

Char. 21: 1 --> 2

Char. 32: 1 --> 2

Char. 37: 2 --> 1

Char. 65: 1 --> 2

Cycloderma_aubryi :

Some trees:

Char. 13: 1 --> 2

Char. 30: 1 --> 2

Char. 46: 1 --> 2

Pelochelys_bibroni :

All trees:

Char. 35: 1 --> 2

Amyda_cartilaginea :

All trees:

Char. 17: 2 --> 1

Char. 40: 3 --> 2

Char. 57: 1 --> 2

Cyclanorbis_elegans :

All trees:

Char. 92: 1 --> 0

Some trees:

Char. 7: 1 --> 0

Char. 8: 1 --> 0

Char. 10: 1 --> 0

Char. 22: 1 --> 2

Char. 23: 1 --> 2

Char. 31: 1 --> 2

Char. 52: 1 --> 2

Char. 55: 1 --> 2

Char. 56: 1 --> 2

Rafetus_euphraticus :

All trees:

Char. 32: 1 --> 2

Char. 57: 1 --> 2

Char. 67: 1 --> 2

Some trees:

Char. 59: 2 --> 3

Apalone_ferox :

All trees:

Char. 59: 2 --> 3

Char. 67: 1 --> 2

Nilssonia_formosus :

All trees:

Char. 0: 3 --> 2

Char. 8: 1 --> 0

Char. 17: 2 --> 1

Char. 21: 2 --> 1

Char. 27: 1 --> 2

Char. 64: 1 --> 2

Cycloderma_frenatum :

All trees:

Char. 44: 1 --> 2

Char. 45: 1 --> 2

Char. 52: 1 --> 2

Char. 55: 1 --> 2

Nilssonia_gangetica :

All trees:

Char. 52: 3 --> 2

Char. 59: 3 --> 2

Char. 60: 2 --> 3

Nilssonia_hurum :

All trees:

Char. 30: 2 --> 3

Char. 60: 2 --> 1

Char. 92: 0 --> 1

Chitra_indica :

All trees:

Char. 2: 2 --> 3

Char. 19: 2 --> 1

Char. 26: 1 --> 2

Char. 29: 1 --> 2

Char. 53: 1 --> 0

Char. 54: 1 --> 0

Char. 64: 1 --> 2

Apalone_mutica :

All trees:

Char. 0: 3 --> 4

Char. 27: 1 --> 2

Char. 52: 3 --> 2

Lissemys_punctata :

All trees:

Char. 93: 0 --> 2

Some trees:

Char. 5: 1 --> 2

Char. 13: 1 --> 2

Char. 79: 0 --> 1

Cyclanorbis_senegalensis :

All trees:

Char. 93: 0 --> 2

Some trees:

Char. 0: 2 --> 3

Char. 5: 1 --> 2

Char. 11: 0 --> 1

Char. 15: 2 --> 3

Char. 17: 2 --> 4

Char. 30: 1 --> 2

Char. 33: 2 --> 1

Char. 80: 1 --> 2

Pelodiscus_sinensis :

All trees:

Char. 0: 3 --> 4

Char. 7: 0 --> 1

Char. 16: 1 --> 3

Char. 27: 1 --> 2

Char. 37: 1 --> 2

Char. 49: 0 --> 2

Apalone_spinifera :

All trees:

No autapomorphies:

Palea_steindachneri :

All trees:

Char. 0: 3 --> 2

Char. 19: 2 --> 1

Char. 28: 1 --> 2

Char. 48: 1 --> 2

Char. 50: 1 --> 2

Char. 55: 2 --> 1

Dogania_subplanus :

All trees:

Char. 0: 3 --> 4

Char. 17: 2 --> 0

Char. 19: 2 --> 3

Char. 41: 1 --> 2

Char. 52: 3 --> 2

Char. 93: 0 --> 2

Trionyx_triunguis :

All trees:

Char. 42: 1 --> 2

Char. 56: 1 --> 2

Some trees:

Char. 54: 1 --> 2

Gilmoremys_lancensis :

All trees:

Char. 27: 1 --> 2

Char. 28: 1 --> 2

Char. 36: 2 --> 3

Char. 80: 1 --> 2

Char. 82: 0 --> 1

Char. 83: 0 --> 1

Plastomenus_thomasii :

All trees:

Char. 80: 1 --> 2

H._rememdium :

Some trees:

Char. 72: 0 --> 1

H._arctochelys :

All trees:

Char. 74: 0 --> 1

Char. 75: 0 --> 1

Some trees:

Char. 72: 0 --> 1

H._tetanetron :

All trees:

Char. 13: 2 --> 1

Some trees:

Char. 86: 1 --> 0

H._sterea :

All trees:

Char. 0: 4 --> 3

Char. 71: 1 --> 0

Helopanoplia_distincta :

All trees:

Char. 79: 0 --> 1

Char. 93: 0 --> 1

Aspideretoides_foveatus :

All trees:

No autapomorphies:

Atoposemys_superstes :

All trees:

Char. 24: 1 --> 2

Char. 71: 0 --> 1

Char. 73: 0 --> 1

Char. 93: 0 --> 1

Perochelys_lamadongensis :

All trees:

No autapomorphies:

Perochelys_hengshanensis_ :

All trees:

No autapomorphies:

Gobiapalone_orlovi :

Some trees:

Char. 0: 3 --> 4

Char. 54: 1 --> 2

Char. 55: 2 --> 1

'Trionyx'_kyrgyzensis :

All trees:

No autapomorphies:

Nemegtemys_conflata :

All trees:

No autapomorphies:

'Aspideretes'_maortuensis :

Some trees:

Char. 18: 3 --> 2

Node 37 :

All trees:

No synapomorphies

Node 38 :

All trees:

Char. 12: 1 --> 2

Some trees:

Char. 14: 1 --> 2

Char. 52: 3 --> 1

Char. 67: 1 --> 2

Node 39 :

All trees:

Char. 4: 2 --> 34

Char. 15: 3 --> 2

Char. 33: 1 --> 2

Char. 40: 1 --> 3

Char. 42: 2 --> 1

Char. 68: 2 --> 1

Char. 76: 1 --> 0

Char. 86: 1 --> 0

Char. 87: 0 --> 1

Char. 91: 0 --> 1

Node 40 :

All trees:

Char. 58: 1 --> 2

Char. 59: 2 --> 3

Char. 61: 1 --> 2

Some trees:

Char. 17: 2 --> 1

Char. 28: 1 --> 2

Char. 30: 3 --> 1

Char. 33: 2 --> 1

Char. 40: 3 --> 2

Char. 47: 1 --> 2

Char. 57: 1 --> 2

Char. 68: 1 --> 2

Node 41 :

All trees:

Char. 3: 1 --> 2

Char. 19: 1 --> 2

Char. 37: 2 --> 1

Char. 78: 1 --> 0

Char. 80: 1 --> 2

Char. 88: 1 --> 0

Node 42 :

All trees:

Char. 0: 2 --> 3

Char. 30: 1 --> 3

Node 43 :

All trees:

Char. 24: 2 --> 1

Char. 30: 3 --> 2

Char. 38: 2 --> 1

Node 44 :

All trees:

Char. 59: 2 --> 3

Char. 63: 1 --> 2

Node 45 :

All trees:

Char. 21: 1 --> 3

Char. 43: 1 --> 2

Node 46 :

All trees:

Char. 16: 1 --> 3

Char. 26: 1 --> 2

Char. 80: 2 --> 1

Node 47 :

All trees:

Char. 55: 2 --> 1

Node 48 :

All trees:

Char. 19: 2 --> 1

Char. 21: 3 --> 2

Char. 49: 0 --> 1

Node 49 :

All trees:

Char. 13: 1 --> 2

Char. 84: 0 --> 1

Node 50 :

All trees:

Char. 0: 3 --> 4

Char. 77: 0 --> 1

Node 51 :

All trees:

Char. 22: 2 --> 1

Char. 70: 0 --> 1

Node 52 :

All trees:

Char. 14: 1 --> 2

Char. 23: 2 --> 1

Char. 76: 0 --> 1

Char. 92: 0 --> 1

Node 53 :

All trees:

Char. 7: 0 --> 1

Char. 71: 0 --> 1

Char. 78: 1 --> 2

Node 54 :

All trees:

Char. 93: 0 --> 2

Node 55 :

All trees:

Char. 70: 0 --> 1

Node 56 :

All trees:

Char. 24: 1 --> 2

Node 57 :

All trees:

Char. 9: 1 --> 0

Char. 19: 2 --> 3

Char. 89: 1 --> 0

Some trees:

Char. 6: 1 --> 2

Char. 10: 1 --> 0

Char. 17: 2 --> 0

Char. 90: 1 --> 0
